# Docosahexaenoic acid counteracts palmitate‐induced endoplasmic reticulum stress in C2C12 myotubes: Impact on muscle atrophy

**DOI:** 10.14814/phy2.13530

**Published:** 2017-12-04

**Authors:** Myra E. Woodworth‐Hobbs, Ben D. Perry, Jill A. Rahnert, Matthew B. Hudson, Bin Zheng, S. Russ Price

**Affiliations:** ^1^ Nutrition and Health Sciences Program Graduate Division of Biological and Biomedical Sciences Emory University Atlanta Georgia; ^2^ Renal Division Department of Medicine Emory University Atlanta Georgia; ^3^ Atlanta VA Medical Center Decatur Georgia; ^4^ University of Delaware Department of Kinesiology and Applied Physiology Newark Delaware; ^5^ Department of Biochemistry and Molecular Biology Brody School of Medicine East Carolina University Greenville North Carolina

**Keywords:** Atrophy, DHA, ER stress, fatty acids, palmitate, skeletal muscle

## Abstract

Lipid accumulation in skeletal muscle results in dysregulation of protein metabolism and muscle atrophy. We previously reported that treating C2C12 myotubes with palmitate (PA), a saturated fatty acid, increases the overall rate of proteolysis via activation of the ubiquitin‐proteasome and autophagy systems; co‐treatment with the omega‐3 polyunsaturated fatty acid docosahexaenoic acid (DHA) prevents the PA‐induced responses. Others have reported that PA induces endoplasmic reticulum (ER) stress which initiates the unfolded protein response (UPR), a collective group of responses that can lead to activation of caspase‐mediated proteolysis and autophagy. Presently, we tested the hypothesis that DHA protects against PA‐induced ER stress/UPR and its atrophy‐related responses in muscle cells. C2C12 myotubes were treated with 500 *μ*mol/L PA and/or 100 *μ*mol/L DHA for 24 h. Proteins and mRNA associated with ER stress/UPR, autophagy, and caspase‐3 activation were evaluated. PA robustly increased the phosphorylation of protein kinase R (PKR)‐like ER kinase (PERK) and eukaryotic initiation factor 2*α* (eIF2*α*). It also increased the mRNAs encoding activating transcription factor 4 (ATF4), spliced X‐box binding protein 1 (XBP1s), C/EBP homologous protein (CHOP), and autophagy‐related 5 (Atg5) as well as the protein levels of the PERK target nuclear factor erythroid 2‐related factor (Nrf2), CHOP, and cleaved (i.e., activated) caspase‐3. Co‐treatment with DHA prevented all of the PA‐induced responses. Our results indicate that DHA prevents PA‐induced muscle cell atrophy, in part, by preventing ER stress/UPR, a process that leads to activation of caspase‐mediated proteolysis and an increase in expression of autophagy‐related genes.

## Introduction

Accumulation of saturated fatty acids (e.g., palmitate) in skeletal muscle causes lipotoxic effects such as cell stress, suppression of insulin signaling, and dysregulation of protein metabolism, all of which directly or indirectly contribute to atrophy (Corcoran et al. 2007; Martins et al. 2012). Treatment of cultured myotubes with palmitate (PA) for 24–48 h results in a significant reduction in myotube diameter, while co‐treatment with the omega‐3 polyunsaturated fatty acid docosahexaenoic acid (DHA) prevents the response (Bryner et al. 2012). Recently, we extended these observations by demonstrating that PA increases the overall rate of protein degradation and that DHA protects against the detrimental effects of PA, in part, by restoring Akt‐mediated inhibition of the FoxO3 transcription factor and subsequent expression of atrogin‐1 E3 ubiquitin ligase and components of the autophagy pathway (i.e., Bnip3, LC3) (Woodworth‐Hobbs et al. 2014).

A high‐fat diet induces endoplasmic reticulum (ER) stress in rodent skeletal muscle and PA has a similar effect on cultured myotubes (Deldicque et al. 2010; Yuzefovych et al. 2013). ER stress leads to activation of a series of coordinated signaling networks called the unfolded protein response (UPR) that act as adaptive responses to re‐establish protein homeostasis (Lee and Ozcan 2014). However, unresolved activation of the UPR can lead to maladaptive responses including caspase‐mediated proteolysis and increased expression of select autophagy proteins. In the case of skeletal muscle, fatty acid‐induced ER stress may serve to provide components of the previously noted proteolytic systems.

Three distinct branches of the UPR system can be initiated by transmembrane effector signal transduction proteins – protein kinase R (PKR)‐like ER kinase (PERK), inositol requiring enzyme 1 alpha (IRE1*α*), and activating transcription factor 6 (ATF6). Early UPR events result in inhibition of global protein synthesis, improved folding capacity of nascent proteins in the ER through selective transcription of a subset of genes, and removal of misfolded or unfolded proteins from the ER via a process called ER‐associated degradation (ERAD) (Dever 2002; Ron and Walter 2007). One such early event, the activation of PERK, reduces the ER's protein load by phosphorylating eukaryotic initiation factor 2*α* (eIF2*α*), reducing the available eIF2•GTP•Met‐tRNA_i_ complex and suppressing global protein synthesis (Harding et al. 1999; Schröder and Kaufman 2005). Mutation of the PERK gene impairs the ability of cells to survive ER stress due to an inability to limit the synthesis of new proteins, suggesting that PERK is required for the adaptive responses to ER stress (Harding et al. 2000). In some cell types, PERK activation has been linked to increases in mRNAs that encode autophagy‐related 12 (Atg12) and Atg5; both proteins participate in autophagosome formation, an early step in the autophagy process (Bernales et al. 2006; Verfaillie et al. 2010; Digaleh et al. 2013). Enhanced autophagy facilitates the removal of damaged ER and insoluble protein aggregates. Whether these responses occur in PA‐treated myotubes is unclear.

Failure to resolve prolonged or severe ER stress can result in activation of caspase‐3 which, in multi‐nucleated muscle cells, acts to degrade myofibrillar and other key proteins involved in cell metabolism. Typically in most cell types, the PERK/eIF2*α* pathway preferentially enhances the expression of activating transcription factor 4 (ATF4) and there are several reports of increased ATF4 gene expression in muscle during diabetes and renal failure (Harding et al. 2000; Lecker et al. 2004; Sacheck et al. 2007). More recently, Ebert et al. (Ebert et al. 2010, 2015) reported that ATF4 is a mediator of fasting‐induced myofiber atrophy and sarcopenia. A key effector gene target of ATF4 is CCAAT‐enhancer binding protein homologous protein (CHOP), another transcription factor (Fawcett et al. 1999; Harding et al. 2000).

Although ATF4 is required for CHOP expression, CHOP is a downstream effector of all three branches of the UPR (Oyadomari and Mori 2004). Enhanced CHOP expression can have several effects on cells including alteration of the balance of pro‐ and anti‐apoptotic proteins that act on mitochondria to control activation (i.e., cleavage) of caspase‐3 (Oyadomari and Mori 2004). Caspase‐3 activation is an important step in the progressive loss of muscle protein due to chronic illness. Inhibition of caspase‐3 attenuates the proteolytic cleavage of actin during diabetes and chronic kidney disease as well as disuse atrophy in the diaphragm (Du et al. 2004; McClung et al. 2007). Relevant to the present study, Peterson et al. (2008) found that PA activates caspase‐3 in myotubes. When considered altogether, these findings led us to posit that PA‐initiated UPR stress may contribute to activation of caspase‐3 activation in muscle cells.

In this study, we investigated the effects of PA on ER stress in myotubes by examining in detail several branches of the ER stress/UPR system. In light of our recent findings that DHA prevents the atrophy‐inducing effects of PA on protein degradation (Woodworth‐Hobbs et al. 2014), we also tested the hypothesis that DHA reduces the PA‐induced activation of ER stress and UPR responses, autophagy‐related gene expression, and caspase‐3 activity.

## Materials and Methods

### Cultured myotube model

Mouse C2C12 myoblasts (American Type Culture Collection, Manassas, VA) were grown in Dulbecco's Modified Eagle Medium (DMEM) containing 4.5 g/L glucose and supplemented with 10% fetal bovine serum (FBS; Atlanta Biologicals, Lawrenceville, GA) plus antibiotics (100 U/mL penicillin, 100 *μ*g/mL streptomycin; Invitrogen, Carlsbad, CA). At 90–95% confluence, cells were induced to differentiate into myotubes in DMEM containing 4.5 g/L glucose plus 2% horse serum (Invitrogen) and antibiotics for 3–4 days before treatment with fatty acids.

### Experimental treatments

Palmitate (PA) and cis‐4, 7, 10, 13, 16, 19‐docosahexaenoic acid (DHA) (Sigma Aldrich, St. Louis, MO) were dissolved in ethanol and added to DMEM containing 2% bovine serum albumin (BSA; Roche, Indianapolis, IN), 2% FBS, 2 mmol/L L‐carnitine (Sigma Aldrich), and 1% antibiotics (treatment media) at final concentrations of 500 *μ*mol/L and 100 *μ*mol/L, respectively. When myotubes were co‐treated with PA and DHA, both fatty acids were added to experimental media which was then added to the cells. Others have reported that this combination of fatty acids and BSA result in a final molar ratio that approximates the levels in human plasma (Santomauro et al. 1999). Control cells were incubated in treatment media with equal amounts of ethanol substituted for PA and DHA. Myotubes were incubated in experimental media for 24 h. In some experiments, thapsigargin (1 *μ*mol/Lol·L) was added to myotubes for 17 h to induce ER stress as described (Deldicque et al. 2011).

### RNA isolation and qPCR analysis

RNA was isolated using TRIzol (Invitrogen) and reverse transcribed using the Superscript III First‐Strand Synthesis kit (Invitrogen) according to the manufacturer's instructions. Specific mRNAs were measured by quantitative real time PCR in a BioRad iCycler with iQ SYBR Green reagent (BioRad Laboratories, Hercules, CA) and previously published primer sets for ATF4 (Ebert et al. 2010), CHOP, XBP1s (Deldicque et al. 2011), Atg5 (Martinez et al. 2011), and Atg12 (De Palma et al. 2012). *β*‐actin was used as the qPCR normalization control. Data were analyzed for fold change (ΔΔC_t_) using the iCycler software, as described (Zheng et al. 2010). Melting curve analyses were performed to verify the specificity of the reaction.

### Western blot analysis

Whole cell lysates were prepared using the buffers specified by the antibody vendors. Protein concentrations of cleared lysates were measured using a BioRad DC protein assay (BioRad Laboratories, Hercules, CA). Western blot analyses were performed as described (Gao et al. 2008) using commercial antibodies to p‐PERK (T980), PERK, p‐eIF2*α*(S51), eIF2*α*, CHOP, cleaved caspase‐3 (Cell Signaling Technology, Beverly, MA), and Nrf2 (Enzo Life Sciences, Farmingdale, NY). Equal protein loading and transfer to blot membranes was verified by Ponceau S Red staining and quantification (Zheng et al. 2010). This method avoids the confounding issues associated with identifying “loading controls” that do not change when the cell treatments being studied alter the synthesis and/or degradation of most cellular proteins.

### Statistical analyses

Data are presented as mean percentage of control ± SE of multiple experiments with 3–4 replicates per experiment. Differences between treatments are compared by one‐way ANOVA with post hoc analysis by Tukey's test for multiple comparisons. For some western blots (i.e., cleaved caspase‐3 and CHOP), bands were undetectable in some lanes even after extensive exposures. In these cases, the data were analyzed using the Freeman‐Halton extension of the Fisher's exact probability test. Results are considered statistically significant at *P* < 0.05. All analyses were performed using GraphPad Prizm or InStat (La Jolla, Ca).

## Results

### DHA attenuates the induction of ER stress by PA

**Figure 1:**
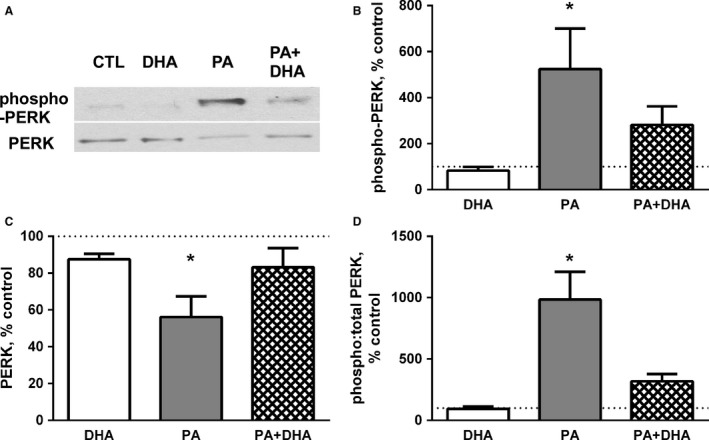
Co‐treatment with DHA attenuates the PA‐induced increase in PERK activation. (A) Representative western blots for phospho‐PERK (T980) and total PERK are shown. (B) PA increases phosphorylation of PERK (*P < 0.05 vs. control and DHA,* n* = 5 experiments) and (C) decreases total PERK (*P < 0.01 vs. control, *n* = 5). (D) Co‐treatment with DHA attenuates the PA‐induced increase in the phospho:total PERK ratio (*P < 0.001 vs. all other groups, *n* = 5). The dotted line across each graph is a reference line for 100% of the mean control value. Data are presented as mean percentage of control ± SE of multiple experiments.

**Figure 2:**
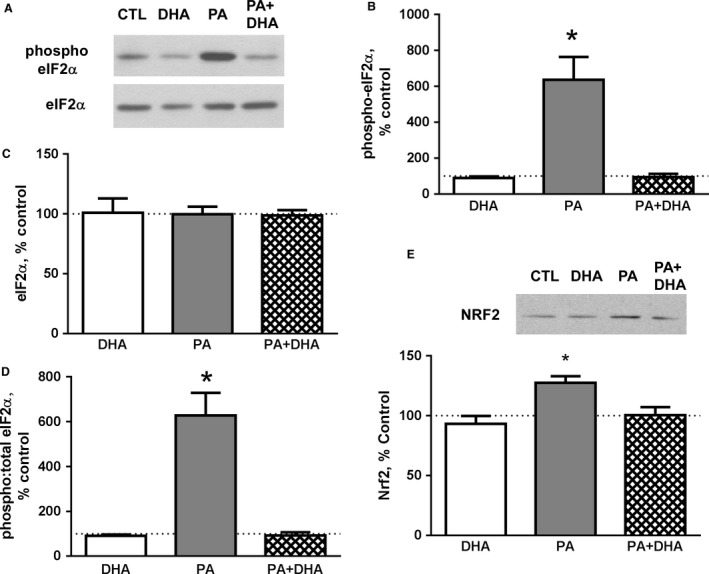
Co‐treatment with DHA attenuates PA‐induced phosphorylation of eIF2*α*. (A) Representative western blots for phospho‐eIF2*α* (S51) and total eIF2*α* are shown. (B) PA increases the phosphorylation of eIF2*α* (*P* < 0.0001, *n* = 4 experiments) while (C) total eIF2*α* protein does not change in response to treatment with the fatty acids (*n* = 4). (D) PA increases the ratio of phospho:total eIF2*α* protein (*P* < 0.0001, *n* = 4), while PA + DHA co‐treatment attenuated the change in phospho:total eIF2*α* protein (*P* < 0.0001, *n* = 4). (E) Co‐treatment with DHA antagonizes the PA‐induced increase in Nrf2 protein (*P < 0.01 vs. all other groups, *n* = 3). A representative western blot is shown above the graph. The dotted line across each graph is a reference line for 100% of the mean control value.

An essential response to ER stress is the PERK‐mediated decrease in global protein synthesis to reduce ER client protein load. ER stress causes dissociation of PERK from the protein chaperone BiP (Lee and Ozcan 2014); subsequent dimerization and trans‐autophosphorylation of its cytosolic domain stimulates its protein kinase activity which can be measured as the phosphorylation of threonine‐980 (T980). In our experiments, treatment with PA for 24 h increased phospho‐PERK (T980) protein by over fivefold (Fig. 1A and B) while total PERK protein was reduced 44 ± 11% (Fig. 1A and C). This resulted in a 9.8‐fold increase in the phospho:total PERK ratio (Fig. 1D). The suppression of global protein synthesis by PERK depends on the deactivation of eIF2 via phosphorylation of the eIF2*α* subunit on serine‐51. Accordingly, PA increased phospho‐eIF2*α* (S51) by ~5.4‐fold (Fig. 2A and B). Total eIF2*α* protein did not change with any fatty acid treatment (Fig. 2A and C). Thus, the phospho:total eIF2*α* ratio was increased by >5‐fold in PA‐treated myotubes (Fig. 2D).

To determine if DHA could prevent PERK activation and relieve the inhibition of eIF2*α*, myotubes were co‐treated with PA and DHA. DHA attenuated both the PA‐induced increase in PERK activation (Fig. 1A–D) and the downstream phosphorylation of eIF2*α* (Fig. 2A–D). To further confirm the effects of DHA on PA‐induced PERK activation, we measured another relevant PERK target, nuclear factor erythroid 2‐related factor (Nrf2). The phosphorylation of Nrf2 by PERK stabilizes it, resulting in an increase in Nrf2 protein (Digaleh et al. 2013). PA augmented Nrf2, and notably, co‐treatment with DHA prevented the response (Fig. 2E).

### DHA attenuates the ER stress‐associated caspase signaling induced by PA

**Figure 3:**
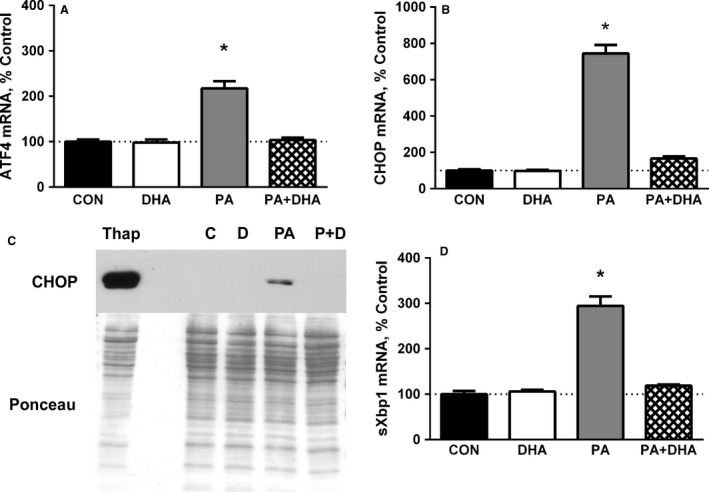
Co‐treatment with DHA antagonizes the PA‐induced increase in UPR‐associated mRNAs and proteins. PA increases (A) ATF4 mRNA (*P < 0.0001 vs. all other groups, *n* = 4 experiments), (B) CHOP mRNA (*P < 0.0001 vs. all other groups, *n* = 4), (C) CHOP Protein (*P* < 0.05 vs. all other groups, *n* = 4; representative western blot shown) and (D) spliced Xbp1 (Xbp1s) mRNA (*P < 0.01 vs. all other groups, *n* = 4). Some cells were treated with 1 *μ*mol/L thapsigargin (Thap) for 17 h to induce ER stress (far left lane). Co‐treatment with DHA prevents all of these responses. The dotted line across each graph is a reference line for 100% of the mean control value.

If ER stress remains unresolved, the UPR transitions from mediating adaptive survival responses to inducing responses associated with autophagy and caspase‐3 (Lee and Ozcan 2014). Typically, these maladaptive responses involve ATF4. In our studies, PA increased ATF4 mRNA by over twofold and DHA prevented the response (Fig. 3A). CHOP is a downstream target of ATF4 as well as the other two arms of the UPR and its transcription is induced in response to severe and/or prolonged ER stress (Oyadomari and Mori 2004). After 24 h, PA robustly and significantly increased the levels of CHOP mRNA (Fig. 3B) and protein (Fig. 3C), while co‐treatment with DHA completely prevented the responses (Fig. 3B and C). Since the basal level of CHOP is typically very low under non‐stressful conditions, it was not surprising that CHOP protein was undetectable in the control or DHA‐treated cells, even after overexposing the films for an extended period of time (Ron and Habener 1992). To test whether the other two arms of the UPR upstream of CHOP were also activated by PA, we measured the level of mRNA encoding the spliced (i.e., active) form of X‐box binding protein 1 (XBP1s), another UPR‐associated transcription factor. It represents the integrated actions of the ATF6 and IRE1*α* pathways; activated ATF6 increases XBP1 transcription whereas the ribonuclease activity of IRE1*α* produces XBP1s (Yoshida et al. 2001). PA significantly increased XBP1s mRNA by almost threefold, and importantly, co‐treatment with DHA prevented the increase (Fig. 3D).

**Figure 4:**
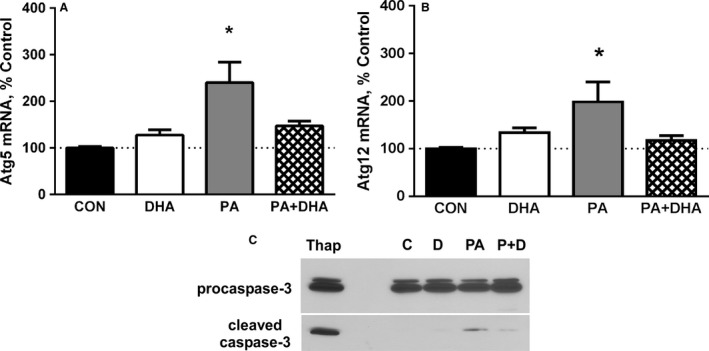
ER stress augments autophagy‐related mRNAs and activates caspase‐3. PA increases Atg5 (A) and Atg12 (B) mRNAs, while co‐treatment with DHA prevents the responses (Atg5, *P < 0.01 vs. all other groups, *n* = 6 experiments; Atg12, *P* < 0.05 vs. control, *n* = 6). The dotted line across each graph is a reference line for 100% of the mean control value. (C) Co‐treatment with DHA (PA+DHA) antagonizes the PA‐induced increase in cleaved (activated) caspase‐3 (*P* < 0.05 vs. all other groups, *n* = 4); no change between groups was detected in procaspase‐3 protein. Myotubes were treated with 1 *μ*mol/L thapsigargin (Thap) for 17 h as a positive control for ER stress/activation of caspase‐3 (far left lane). A representative western blot is shown; the top and bottom panels were taken from the same blot after exposure to film for different lengths of time with the bottom panel exposed for a longer time.

Induction of CHOP signaling can result in several cellular responses including activation of autophagy and caspase‐3 (Heath‐Engel et al. 2008). In some cell types, CHOP and/or its upstream mediator, ATF4, increase the transcription of genes involved in autophagy (e.g., Atg5, Atg12) (B'chir et al. 2013). Since expression of two autophagy‐related proteins, Bnip3 and LC3b, was increased by PA in our earlier study (Woodworth‐Hobbs et al. 2014), we measured Atg5 and Atg12 mRNAs to determine if PA also increases their levels in our experimental model. Atg5 mRNA was increased 240% by PA (*P* < 0.01) and was normalized by co‐treatment with DHA (Fig. 4A). Similarly, PA increased Atg12 mRNA 198% and DHA plus PA prevented the increase (Fig. 4B). To test for caspase‐3 activation, we evaluated protein samples for the presence of cleaved (i.e., activated) caspase‐3. Like CHOP, cleaved caspase‐3 is normally absent or present at a low level in unstressed cells. As expected, cleaved caspase‐3 was undetectable in the control and DHA‐treated samples, whereas PA induced a significant increase in caspase‐3 cleavage and co‐treatment with DHA attenuated the response (Fig. 4C).

## Discussion

**Figure 5:**
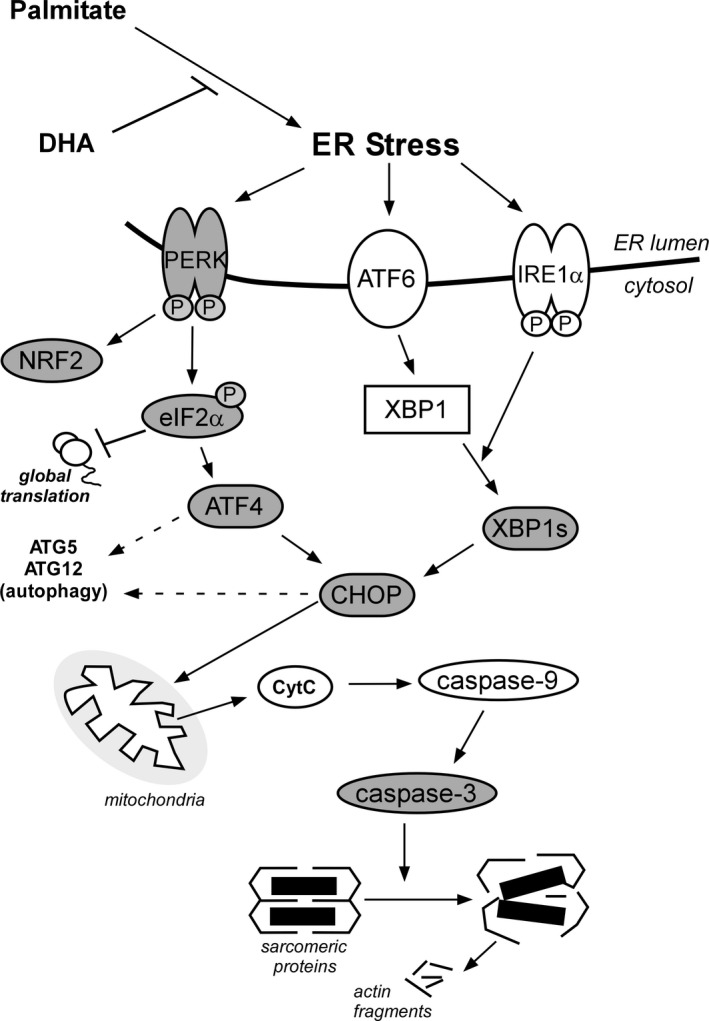
ER stress and the caspase proteolytic system are important regulators of muscle protein homeostasis. PA induces unfolded protein responses by activating all three arms of the ER stress system. These responses lead to activation of caspase‐3, presumably by inducing the intrinsic caspase‐3 pathway, and by increasing the expression of genes involved in autophagy. DHA counteracts the effects of PA by reducing ER stress, thus antagonizing the increase in UPR mRNAs and proteins and preventing caspase‐3 cleavage. Signaling and effector mRNAs/proteins investigated in this study are indicated in gray.

The ER is responsible for folding, processing, trafficking, and quality control of many cellular proteins. In response to cell stress, it can both suppress the general synthesis of nascent proteins and augment the activity of several proteolytic systems (e.g., ubiquitin‐proteasome, autophagy) that remove damaged proteins and organelles (Heath‐Engel et al. 2008; Lee and Ozcan 2014). Persistent activation of ER stress induces UPR signals that can lead to mitochondrial instability and activation of caspase‐mediated proteolysis and/or autophagy‐related gene expression (Ron and Walter 2007; Heath‐Engel et al. 2008). All three of these proteolytic systems are well‐documented to be involved in the loss of muscle mass during chronic illness (Price et al. 2010; Sandri 2013). We recently reported that PA reduces the cross‐sectional area of C2C12 myotubes and increases their measured rate of protein degradation, in part, by enhancing FoxO3 transcription factor activity via reduced Akt activity (Bryner et al. 2012; Woodworth‐Hobbs et al. 2014). This results in upregulation of the ubiquitin‐proteasome system and some components of the autophagy pathway. Importantly, co‐treatment of DHA with PA fully restored Akt signaling as well as the rate of protein degradation in those studies. Our present results extend these earlier findings by examining the effects of PA and DHA on other key intracellular pathways and components of the protein degradation process in skeletal muscle. Specifically, we demonstrate that DHA also prevents the induction of the caspase‐3 protease and Atg5/Atg12 mRNAs by PA (Fig. 5). To the best of our knowledge, our results are the first to establish: 1) a link between atrophy‐related responses (i.e., proteolytic system mRNAs and proteins) and PA‐induced activation of multiple branches of the UPR; and 2) a protective role of DHA on ER stress/UPR and proteolysis‐related responses in skeletal muscle.

Similar to Deldicque et al. (2010) who studied skeletal muscles of mice fed a high‐fat diet, treating myotubes with PA for 24 h potently activates PERK, as indicated by its increased phosphorylation. PERK activation typically leads to the phosphorylation of Ser‐51 in eIF2*α* which “reprograms” its function in the protein translation process (Baird and Wek 2012). This change leads to a decrease in the global rate of protein synthesis which reduces ER client protein load while also preferentially inducing translation of a subset of proteins involved in the stress response. Consistent with the observed PERK response, PA induces the phosphorylation of eIF2*α* and increases the level of Nrf2, another target of PERK that is stabilized by phosphorylation. Importantly, the increase in phospho‐PERK, phospho‐eIF2*α*, and Nrf2 were all reversed with DHA co‐treatment, findings consistent with the conclusion that DHA decreases PERK activation and the inhibitory reprogramming of eIF2*α*.

In myotubes, ER‐stress‐related changes in components of the UPR can result in mitochondrial instability and activation of both caspase proteases and autophagy. In several models of muscle atrophy, ATF4 appears to mediate some of these responses (Harding et al. 2000). PERK/eIF2*α* signaling enables the preferential increase in ATF4 protein by selectively enhancing both the transcription of the *ATF4* gene and subsequent translation of the ATF4 mRNA (Dey et al. 2010). Our result documenting a PA‐induced increase in ATF4 mRNA is consistent with this earlier finding.

Although ATF4 is essential for the induction of CHOP transcription, the CHOP promoter also contains binding sites for ATF6 and XBP1s and all three transcription factors are required for maximal induction of CHOP (Yoshida et al. 2001; Oyadomari and Mori 2004). Since XBP1s mRNA represents an integrated readout of the ATF6 and IRE1*α* pathways and PA robustly increases the level of XBP1s mRNA as well as CHOP mRNA and protein, our data indicate that all three branches of the UPR are activated by the fatty acid. Furthermore, DHA prevents the activation of all three UPR arms.

The current results build on our earlier findings by providing evidence that additional mechanisms (i.e., ER stress/UPR) act to support the PA‐augmentation of protein degradation (Woodworth‐Hobbs et al. 2014). Since Nrf2 increases the expression of several proteasome subunits, an increase in Nrf2 protein, such as that seen with PA treatment, is an indicator of increased ERAD and ubiquitin‐proteasome system activities (Digaleh et al. 2013). PA also increases the levels of Atg5 and Atg12 mRNAs, consistent with the observed up‐regulation of other mRNA and proteins (i.e., Bnip3, LC3) involved in autophagosome formation (Woodworth‐Hobbs et al. 2014). In addition, Atg5 and Atg12 can also act in concert with CHOP at the level of mitochondria to activate caspase‐3 and we found evidence consistent with this as well (Yousefi et al. 2006; Heath‐Engel et al. 2008; Rubinstein Assaf et al. 2011; Wing et al. 2011). Importantly, activation of caspase‐3 in multi‐nucleated myotubes and myofibrils typically does not result in classical apoptosis (Borisov and Carlson 2000). Instead, caspase‐3 contributes to the regulation of muscle protein metabolism in multiple ways. It facilitates the degradation of myofibrillar and other muscle proteins by producing fragments that are degraded by the ubiquitin‐proteasome system (Du et al. 2004; Workeneh et al. 2006). It also enhances proteasome activity through cleavage of several 19S subunits (Wang et al. 2010). In addition, caspase‐3 may reshape protein synthesis by altering the activities of several protein translation factors through proteolytic cleavage (Marissen et al. 2000).

In a recent study, Bohnert et al. (2016) investigated for the first time whether 4‐phenylbutyrate (4‐PBA), a chemical chaperone that attenuates ER stress, would reduce the muscle atrophy induced by cancer (Özcan et al. 2006; Hu et al. 2014). Curiously, they found that 4‐PBA induced atrophy responses when administered to mice or cultured myotubes; it also exacerbated the wasting induced by tumors in mice and the reduction in diameter of cultured myotubes incubated in tumor‐conditioned media. These surprising effects of 4‐PBA could reflect a response to severe, prolonged inhibition of all three arms of ER stress; mice were treated for 18 days and myotubes were treated for 24 h. 4‐PBA also has been reported to inhibit effector proteins like HDAC5, raising the possibility that the observed responses represent effects unrelated to ER stress (Hu et al. 2014). Moreover, these outcomes are in contrast to those of Ebert et al. (2015) who used small molecule inhibitors of ATF4 to show its important role as a mediator of age‐ and fasting‐related atrophy. Nonetheless, the role of ER stress in muscle atrophy is an area of research that warrants more extensive examination to understand these differences.

In summary, our study provides additional evidence that the signaling pathways which coordinate protein degradation and protein synthesis are complex, extensive, and interconnected. Specifically, the process by which elevated saturated fatty acids cause muscle loss is multi‐faceted and involves coordination of multiple signaling pathways and proteolytic systems. Our current results demonstrate that PA induces multiple branches of the UPR in cultured myotubes. This leads to increased expression of genes encoding components of the autophagy system as well as activation of the caspase‐3 proteolytic system. Both of these systems contribute to the muscle loss that occurs in a variety of diseases, many of which are linked to hyperlipidemia and insulin resistance (e.g., type II diabetes, chronic kidney disease, and heart failure) (Franch and Price 2005; Carrero et al. 2013). Importantly, DHA provides protection against nearly all of the atrophy‐inducing effects of PA identified to date. Although its mechanism(s) of action remains unclear, its anti‐proteolytic effects impact multiple signaling pathways (e.g., Akt/FoxO, UPR). Such multi‐faceted properties suggest that nutritional supplements enriched in DHA could be a useful therapy to attenuate muscle loss linked to many wasting conditions. Indeed, omega‐3 fatty acid supplements (i.e., DHA, EPA) have been reported to provide promising muscle‐sparing effects against disease‐associated cachexia and sarcopenia (Ma et al. 2015; Pappalardo et al. 2015; Smith et al. 2015).

## Conflict of Interest

J.A.R., B.Z., B.D.P., M.B.H., M.E.W.H., and S.R.P.; no conflict of interest disclosures. The contents do not represent the views of the U.S. Department of Veterans Affairs or the United States Government.
